# High level expression of human epithelial β-defensins (hBD-1, 2 and 3) in papillomavirus induced lesions

**DOI:** 10.1186/1743-422X-3-75

**Published:** 2006-09-08

**Authors:** Kong T Chong, Liangbin Xiang, Xiaohong Wang, Eunjoo L Jun, Long-fu Xi, John M Schweinfurth

**Affiliations:** 1 Department of Otolaryngology & Communicative Sciences, University of Mississippi Medical Center, Mississippi, USA; 2 Department of Microbiology, University of Mississippi Medical Center, Mississippi, USA; 3 Department of Psychiatry, University of Mississippi Medical Center, Mississippi, USA; 4 Department of Pathology, University of Washington School of Medicine, Washington, USA

## Abstract

**Background:**

Epithelial defensins including human β-defensins (hBDs) and α-defensins (HDs) are antimicrobial peptides that play important roles in the mucosal defense system. However, the role of defensins in papillomavirus induced epithelial lesions is unknown.

**Results:**

Papilloma tissues were prospectively collected from 15 patients with recurrent respiratory papillomatosis (RRP) and analyzed for defensins and chemokine IL-8 expression by quantitative, reverse-transcriptase polymerase chain reaction (RT-PCR) assays. HBD-1, -2 and -3 mRNAs were detectable in papilloma samples from all RRP patients and the levels were higher than in normal oral mucosal tissues from healthy individuals. Immunohistochemical analysis showed that both hBD-1 and 2 were localized in the upper epithelial layers of papilloma tissues. Expression of hBD-2 and hBD-3 appeared to be correlated as indicated by scatter plot analysis (r = 0.837, p < 0.01) suggesting that they were co-inducible in papillomavirus induced lesions. Unlike hBDs, only low levels of HD5 and HD6 were detectable in papillomas and in oral mucosa.

**Conclusion:**

Human β-defensins are upregulated in respiratory papillomas. This novel finding suggests that hBDs might contribute to innate and adaptive immune responses targeted against papillomavirus-induced epithelial lesions.

## Background

Recurrent respiratory papillomatosis (RRP) is a disease associated with human papillomavirus (HPV) infection of the upper respiratory tract [1,2]. The condition is characterized by abnormal proliferation of epithelial keratinocytes leading to papilloma formation, most commonly in the larynx. The disease is often diagnosed during childhood and some patients have recurrent lesions throughout adulthood. RRP is associated with significant morbidity and can be life- threatening because of airway obstruction. Current medical treatments are unsatisfactory and repeated surgeries are required to relieve symptoms [3].

The pathogenesis of respiratory papilloma is poorly understood. Although RRP is a relatively rare disease, HPV infection is not uncommon in normal oral mucosa [4]. It is thought that host factors such as immunodeficiency may predispose susceptible individuals to reactivation of HPV infection [5,6]. Hence, both cell-mediated and humoral immune mechanisms have been widely investigated for their roles in HPV infection and disease [7,8]. In contrast, much less is known about the role of innate immunity in HPV infection, even though HPV disease is characterized by localized viral replication and lesion formation in the mucosal or cutaneous epithelial cells [8,9]. Since papillomaviruses complete their replication cycle in terminally differentiated cells and release progeny virions through desquamation of the epithelial surface, there is relatively little exposure of viral antigens to the mechanisms of immune surveillance. Therefore, infection with HPV tends to be more persistent than with other microbes. However, the vast majority of HPV infections in immune competent hosts are eventually resolved, as evidenced by the high rate of remission of primary genital HPV infection. This suggests that most infected hosts are capable of mounting an effective immune response against HPV infections.

Recent understanding of innate immunity indicates that in addition to providing a first-line of defense against invading organisms, innate immune mechanisms also trigger the adaptive immune response [9]. Some important components of innate mucosal immunity are α and β defensins, which are cysteine-rich, cationic peptides that display broad-spectrum antimicrobial activity. Although α-defensins (HD1, -2, -3) are predominantly found in leukocytes, HD5 and HD6 are expressed in intestinal and genital tract epithelia [9]. Human β-defensins (hBDs) including hBD-1, hBD-2, and hBD-3 are widely expressed in epithelial cells [9,10]. These are detectable in most cutaneous and mucosal sites including the normal airway and oral epithelium and are believed to be key mediators of innate mucosal defense system [11-15]. For example, both constitutively expressed hBD-1 and induced hBD-2 and hBD-3 have been shown to be microbicidal to a variety of bacterial and fungal pathogens [16,17], and more recently, shown to inactivate both enveloped and non-enveloped viruses including human immunodeficiency virus type 1 (HIV-1) and adenovirus [18,19]. In addition to their antimicrobial activity, hBD-2 also links innate and adaptive immunity by attracting memory T cells and recruiting immature dendritic cells through chemokine receptor CCR6 [20].

Due to their location and role in antigen presentation, epithelial dendritic or Langerhans cells are believed to be essential for the initiation of adaptive immune response against HPV infection [21]. Dendritic cells have been shown to interact with HPV virions and virus-like-particles (VLP) and thus play a role in inducing protective immunity against primary HPV infection [22-24]. Hence, the demonstrated role of dendritic cells in HPV immunity together with the influence of hBDs on their recruitment makes it important to investigate the role of hBDs in papillomavirus infections. We hypothesized that as with other infections, expression of hBDs might be elevated in HPV infected tissue and that hBDs might facilitate host defense against papillomavirus infection.

We therefore investigated the expression of epithelial associated defensins (hBD-1, hBD-2, hBD-3, HD5 and HD6) in papilloma specimens obtained from RRP patients and in normal oral mucosa. Since interleukin-8 (IL-8) or CXC-chemokine ligand 8 (CXCL8) is produced by epithelial cells in response to infection or inflammatory stimulation [25], we also investigated IL-8 expression in these patients in order to determine if IL-8 is upregulated in papillomas and whether defensin expression is associated with IL-8 expression. We also attempted to relate IL-8 and defensin expression to patient characteristics including HPV genotype and disease severity.

## Results

### RRP patient demographics

The study population consisted of 15 patients with recurrent disease from both urban and rural areas in the state of Mississippi. Demographics including racial background, age, sex and disease severity are shown in Table 1. These patients were divided into a juvenile group (age range 3–12 years), and an adult group (age range of 24 to 70 years). The diagnosis of RRP was determined by routine histopathology and clinical criteria with the disease severity categorized as mild or severe (Table 1). HPV detection and typing were performed for each patient so that we could analyze the effect of infection with specific HPV type on the expression of IL-8 and defensins. By using a reverse line blot assay that could identify 37 HPV genotypes, we found that all patients were positive for HPV-6 or 11. It is interesting to note that all 9 patients in the juvenile group were infected with HPV-11 and 4 of the patients demonstrated co-infection with HPV-6. In the adult group, only 1 of 6 patients showed co-infection with HPV-6 and 11; the rest were either infected with HPV-11 or HPV-6 (Table 1).

**Table 1:** Patient demographics and HPV types

Patient No.	Sex	Age	Race	Disease state	HPV type
Juvenile					
1	Female	10	AA	Severe	11
2	Female	3	AA	Severe	11
3	Female	5	C	Severe	6,11
4	Female	6	C	Severe	6,11
5	Female	3	AA	Severe	11
6	Male	5	AA	Severe	6,11
7	Female	12	AA	Mild	11
8	Female	10	AA	Severe	6,11
9	Male	8	AA	Severe	11
					
Adult					
10	Male	57	C	Severe	6,11
11	Female	35	AA	Mild	11
12	Male	32	AA	Mild	11
13	Female	24	C	Mild	6
14	Male	70	C	Mild	6
15	Male	45	C	Mild	11

**NOTE**. AA, African American. C: Caucasian

### Expression of hBD-1, hBD-2, hBD-3, HD5 and HD6 mRNA in papilloma tissues

**Figure 1:**
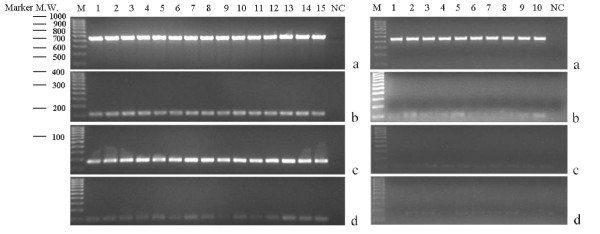
RT-PCR analysis of hBD-1, hBD-2 and hBD-3 mRNA expression in RRP papilloma samples from 15 different individuals ((Left Panel, Lanes 1–15) and normal oral mucosa tissue from 10 different individuals (Right Panel, Lanes 1–10). A housekeeping gene, β-actin (a), was detected as a 450 bp PCR product; hBD-1 (b), hBD-2 (c) and hBD-3 (d) expressions were detected as 108, 172 and 98 bp PCR products, respectively. Samples were separated by 2% agarose gel electrophoresis and stained with ethidium bromide. 100 bp-ladder molecular-weight markers are presented on the left of the panel. Negative controls including amplification without reverse transcriptase or with cDNA sample replaced by DNase, RNase – free, distilled water were performed in each experiment but are not shown due to limited gel space.

**Figure 2:**
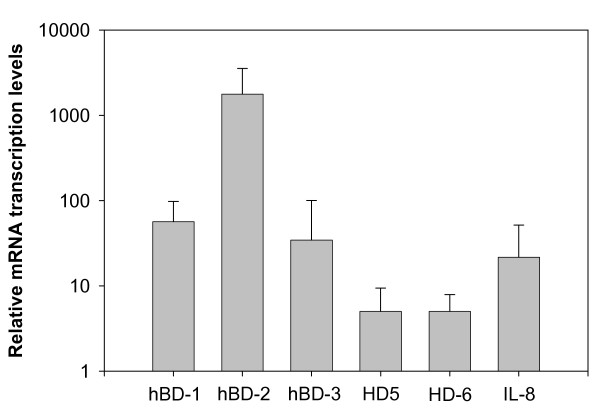
hBD-1, hBD-2, hBD-3, HD5, HD6 and IL-8 mRNA expressions were analyzed by real-time RT-PCR. Bars represent defensins expression normalized to β-actin and relative to normal mucosa using the 2^-ΔΔCT ^analysis as described in Materials and Methods. Error bars represent the standard error of the mean of triplicate analysis.

Defensin expression was determined in freshly-obtained papilloma specimens from 15 patients undergoing surgical treatment. hBD-1 and -2 were readily detected in all papilloma tissue samples by RT-PCR analysis (Figure 1). hBD-3 expression was weaker but still detectable in papilloma samples from all RRP patients. In similar experiments, hBD-1, hBD-2 and hBD-3 were also detectable in all ten samples of normal oral mucosa (Figure 1). The expression of hBDs was quantified by real-time PCR and hBD expression relative to normal oral mucosa is shown in Figure 2. Expression of hBD-2 was highly upregulated as evident by >1000-fold higher relative transcript levels. Although hBD-3 was clearly expressed in papilloma tissues, its level was much lower than either hBD-1 or hBD-2 (Figure 2). Unlike the hBDs, HD5 and HD6 were not consistently detectable at 35 cycles of PCR amplification but were measurable at 40 cycles of amplification as performed for real-time PCR (Figure 2).

### Immunolocalization of hBD-1 and hBD-2 in papilloma tissue sections

**Figure 3:**
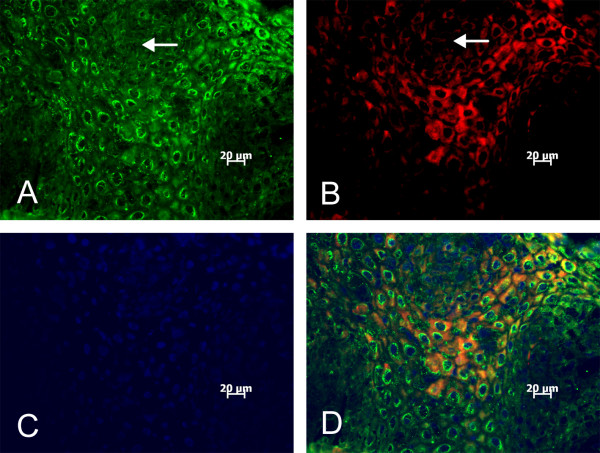
Immunostaining of a representative frozen section of respiratory papilloma with antibody preparations to hBD-1 or hBD-2. Defensins were detectable as strong perinuclear staining of hBD-2 (A) and cytoplasmic immunostaining of hBD-1 (B) in all papilloma sections. Tissue section was counterstained with DAPI (C), and a merged picture was shown in (D). Arrows indicate areas of negative staining for hBD-1 and hBD-2 immunostaining. Tissue sections exposed to antibody preparations derived from preimmune or unrelated immunogens showed no reactivity (data not shown).

To determine defensin protein expression, frozen sections of respiratory papilloma tissues were subjected to immunohistochemical staining with polyclonal rabbit antiserum for hBD-1 and goat antiserum for hBD-2. Fluorescence microscopy showed strong immunostaining of hBD-1 and hBD-2 in the upper epithelial layers including the stratum granulosum and stratum spinosum (Figure 3). Unlike hBD-1, hBD-2 staining was more granular and was strongly perinuclear (Figure 3A). Some epithelial cells were stained for both hBD-1 and hBD-2, but hBD-2 staining appeared to be more widespread, especially in spinous layers. Staining appeared to be specific for hBDs since tissue sections exposed to serum preparations derived from preimmune or unrelated immunogens showed no reactivity (data not shown).

### Correlation of mRNA expression among hBDs and IL-8 in papilloma tissues

IL-8 mRNA was expressed at low levels in normal mucosa in healthy individuals but was modestly upregulated in papilloma samples from RRP patients (Figure 2). As expected of the inducible hBDs, hBD-2 expression was highly correlated with hBD-3 as shown by scatter plot analysis (r = 0.837, p < 0.001). In our small study population, we did not identify a correlation between hBD expression and patient characteristics such as age, gender and racial background. Expression of hBDs also appeared not to correlate with infection with specific HPV types or lesion severity. However, there was a trend that patients with "severe" disease progression were more likely to display higher levels of IL-8 expression compared to "mild" progression disease group (r = 0.537, p = 0.039).

## Discussion

This report is the first to identify upregulated expression of epithelial defensins (hBD-1, -2 and -3) in papillomavirus associated lesions. RRP is a rare disease and therefore our study population is relatively small. However, our results clearly demonstrate the expression of epithelial defensin in 100% of patient-derived papilloma tissue samples. The modest but consistent upregulation of hBD-1 in papillomas is unusual because hBD-1 expression is predominantly constitutive in most tissues. However, upregulated expression of hBD-1 has been reported in monocytes exposed to interferon-γ, bacteria or endotoxin [9], and in human uterine epithelial cells exposed to poly (I:C), an agonist for Toll-Like-Receptor 3 [26]. Our findings suggest that, as with other microbial pathogens, infection with papillomavirus is associated with high levels of epithelial defensins even though HPV induced lesions are not thought to elicit significant levels of tissue inflammation.

Although our study was performed on laryngeal papillomas, hBD expression is likely upregulated in other papillomavirus-induced lesions including both cutaneous and mucosal genital warts since these lesions share very similar pathology. Several defensins, including hBD-1, hBD-2 and HD5, are known to be expressed in epithelial cells of the urogenital tract [9]. Therefore, it is likely that these epithelial defensins may also play a role in genital HPV infection. However, HD5 and HD6 are not associated with RRP since these are only minimally expressed in respiratory papilloma tissues. Others have reported that HD5 is primarily expressed in intestinal paneth cells and the female genital tract [9], but appears to be absent in airway epithelia [27].

Recently, it has been reported that some members of the defensin family of peptides play important roles in host defense against certain viral infections such as the human immunodeficiency virus (HIV-1), influenza and herpesviruses [18,28-31]. In addition to directly inactivating virion infectivity, defensins have been reported to block HIV-1 viral replication in CD4+ cells by the inhibition of PKC signaling [30]. In contrast, very little is known about the potential role of defensins in HPV infection. Nevertheless, several α-defensins (but apparently not the epithelial β-defensins) have recently been shown to inhibit the initial stages of HPV replication in a pseudovirus assay system [32]. However, unlike HD5 that is expressed in the genital tract, leukocyte associated α-defensins are much less likely to be involved in HPV infection because of the limited cellular infiltration in HPV lesions. In our study, hBDs appeared to be ineffective at limiting viral disease progression since papillomas persisted despite the high level expression of hBDs. Although we have not studied the time course of hBDs upregulation in relation to infection, it is tempting to speculate that hBDs might serve more as signaling molecules that facilitate the generation of adaptive immune responses against a virus infection that elicits little inflammatory events.

## Conclusion

We demonstrated that beta-defensins were upregulated in respiratory papillomas. The presence of inducible defensins suggests that defensins might contribute to innate and adaptive immune responses targeted against papillomavirus infection. This observation is relevant to vaccine development and could provide a rationale for the development of defensin-based therapy for HPV using exogenous defensin preparations or by enhancing the production of defensins in target epithelial surfaces.

## Methods

### Patient population and specimen collection

The study protocol and all tissue procurement procedures were reviewed and approved by the University of Mississippi Medical Center Institutional Review Board overseeing research on human subjects. Fifteen patients undergoing surgical treatment for RRP at a tertiary care center between January 2004 and January 2005 were included in the study. Written, informed consent specifying the use of remaining tissue was obtained from each patient or legal guardian prior to collection. For control experiments, samples of oral mucosa were collected from ten individuals undergoing tonsillectomy due to tonsillar hypertrophy. These patients had no symptoms of acute, non-HPV upper-respiratory infection such as dysphagia, sore throat, elevated body temperature, signs of inflammation or plugs in the surface of the palatine tonsil. At the time of surgery, they were not under drug treatment and had no history of recurrent tonsillitis or allergy. Based on the findings at the time of surgery, RRP patients were diagnosed as mild or severe disease. We defined "Mild" as disease confined to one surface of the larynx or recurrence less than every six months. "Severe" includes patients with lesions in any part of the larynx that may completely fills the glottic airway or spreads outside of larynx and with recurrence between one to six months.

### Histology and immunohistochemistry

Tissue specimens were fixed in 10% buffered formalin and processed for routine histological analysis. Frozen sections were cut at 6 μM thickness from tissue samples that were embedded in OCT-type freezing compound and snap-frozen in liquid nitrogen. For immunostaining, rabbit polyclonal antiserum specific for hBD-1 and goat antibody specific for hBD-2 were obtained from Santa Cruz Biotechnology, Inc. (Santa Cruz, CA). For double labeling, FITC-labeled, donkey anti-goat and Texas red-labeled, goat anti-rabbit antibodies were used. Also, mounting medium (Vector Laboratories, Burlingame, California) with DAPI was used for counterstaining.

### DNA isolation and HPV typing

Total DNA was extracted using the Gentra Systems Puregene DNA Purification Kit (Minneapolis, Minn). Specimens were tested for the presence of HPV using improved PGMY 09/11 L1 consensus primer systems (a set of 5 upstream oligonucleotides comprising the PGMY 11 primer pool and a set of 13 downstream oligonucleotides comprising PGMY 09 primer pool) which amplify a 450 bp fragment of the L1 open-reading frame of a broad spectrum of HPV genotypes [33]. Amplifications were performed using the following conditions: 95°C for 10 mins and 40 cycles of denaturation at 95°C for 1 min, annealing at 55°C for 1 min, and extension at 72°C for 1 min, then followed by final extension at 72°C for 5 min and a hold step at 4°C. To assess tissue integrity, human β-globin gene was co-amplified along with the HPV consensus primers. The PCR products were separated by electrophoresis on 2% agarose gels and visualized by ethidium bromide staining. The HPV genotypes in PCR products were determined using Roche HPV Consensus PCR/Line Blot Genotyping [34] reagents according to manufacturer's protocol.

### Total RNA isolation

Tissue samples for RNA extraction were collected and stored in RNAlater buffer (Ambion) and total RNA was extracted using TRIzol Reagent (Invitrogen) according to manufacturer's protocol. The RNA preparation was dissolved in 50–100 μl RNase free water depending on the size of pellet. Residual DNA was removed from RNA preparations by digestion with DNase Treatment and Removal Reagents (DNA-*Free*, Ambion). The concentration and purity of RNA samples were determined spectrophotometrically by measuring absorbance at 260 and 280 nm using NanoDrop 1000 A Spectrophotometer.

### RT-PCR

Two μg of total RNA was reverse transcribed in a solution of 20 μl containing 50 ng random hexamer primers, 1 mM dNTP mix, 2 μl 10 × RT-buffer, 5 mM MgCl_2_, 10 mM DTT, 40 U RNaseOUT, and 200 U SuperScript III (Invitrogen) reverse transcriptase at 25°C for 10 min, 50°C for 50 min and the reaction was terminated at 85°C for 5 min. Control reactions were set up lacking reverse transcriptase to assess the level of contaminating genomic DNA. RNA template was removed from the cDNA: RNA hybrid by incubation with RNase H. The synthesized cDNA was then amplified by PCR using specific sense and antisense primers for the genes of interest along with a housekeeping gene, β-actin, as control. Primers (Invitrogen) used were as follows: hBD-1 sense 5'-CCTTCTGCTGTTTACTCTCTGC-3', antisense 5'-CCACTGCTGACGCAATTGTAATG-3'; hBD-2 sense 5'-ATCAGCCATGAGGGTCTTGT-3', antisense 5'-GAGACCACAGGTGCCAATTT-3'; hBD-3 sense 5'-CTTCTGTTTGCTTTGCTCTTCC-3', antisense 5'-CCTCTGACTCTGCAATAATA-3'; HD5 sense 5'-ACCTCAGGTTCTCAGGCAAGAGC-3', antisense 5'-GACACAAGGTACACAGAGTAAAATGT-3'; HD6 sense 5'-GCTTTGGGCTCAACAAGGGCTTTC-3', antisense 5'-GACACACGACAGTTTCCTTCTAGGTCATA-3'; IL-8 sense TTGGCAGCCTTCCTGATTTC-3', antisense 5'-AACTTCTCCACAACCCTCTG-3', and β-actin sense 5'-TGTGCCCATCTACGAGGG GTATGC-3', antisense 5'-GGTACATGGTGGTGCCGCCAGACA-3'). Thermal cycling conditions consisted of an initial denaturing step (96°C, 5 min) followed by 35 cycles of denaturing (94°C, 30 s), annealing (30 s at temperatures for specific primers as stated below), and extending (72°C, 30 s), followed by 3 min at 72°C for elongation. The annealing temperature for hBD-1, hBD-2, hBD-3, HD5, HD6, IL-8 and β-actin were 52°C, 52°C, 43°C, 53°C, 61°C, 49°C, and 64°C, respectively. RT-PCR products were subsequently verified by electrophoresis on 2% agarose gels containing 0.5 μg/ml ethidium bromide and visualized under UV transillumination. Molecular weights of the products were determined using a DNA molecular-weight marker (Perfect DNA 100 bp Ladder, Novagen, Madison WI). Under these conditions, PCR products of 108 bp (hBD-1), 172 bp (hBD-2), 98 bp (hBD-3), 251 bp (IL-8) and 450 bp (β-actin) were generated. All amplification products detected in this study were sequenced to confirm the identity of the defensin of interest.

### Real-time quantitative PCR analysis

Real-time quantitation of defensin mRNA was performed using SYBR-green PCR assay and an iCycler PCR machine (Bio-Rad Laboratories, Hercules, CA). 0.5 μl cDNA was amplified in a 25 μl reaction solution containing 22.5 μl of iQ SYBR Green supermix (Bio-Rad) and 1 μl of each primer (as described above). Each sample was loaded in triplicate and run at 40 cycles under the conditions stated above. After each run, melting curves were generated to confirm amplification of specific transcripts. To determine relative levels of gene expression, the comparative threshold cycle (C_T_) method was employed [35]. C_T _was defined as the cycle number at which reporter fluorescence reached 10 times the standard deviation of the baseline fluorescence. For each sample, the mean C_T _value obtained for β-actin was subtracted from the mean C_T _value for the gene of interest to derive a ΔC_T _value. The ΔC_T _of test samples was then subtracted from ΔC_T _of the control sample to generate a ΔΔC_T_. The mean of these ΔΔC_T _measurements was then used to calculate target gene expression normalized to β-actin and relative to the control as: Relative Expression = 2^-ΔΔCT^.

### Statistics

The relationships between mRNA expression of hBD-1, hBD-2, hBD-3 and IL-8 in RRP subjects (n = 15) were studied using the scatter plot method. Statistical comparisons were computed using Spearman's nonparametric correlations for each bivariate pair.

## Competing interests

The author(s) declare that they have no competing interests.

## Authors' contributions

LX, ELJ and XW performed the laboratory studies. LFX assisted with HPV typing. JMS performed the clinical work, recruitment of patients, and procurement of specimens. KTC conceived of the study, and participated in its design and coordination and drafted the manuscript. All authors read and approved the final manuscript.

## References

[B1] Abrahamson AL, Steinberg BM, Winkler B (1987). Laryngeal papillomatosis: Clinical, histologic and molecular studies. Laryngoscope.

[B2] Kahima HK, Mounts P, Shah K (1996). Recurrent respiratory papillomatosis. Obstet Gynecol Clin North Am.

[B3] Kimberlin DW (2004). Current status of antiviral therapy for juvenile-onset recurrent respiratory papillomatosis. Antiviral Research.

[B4] Praetorius F (1997). HPV-associated diseases of oral mucosa. Clin Dermatol.

[B5] Bonagura VR, Hatam L, DeVoti JA, Zeng F, Steinberg BM (1999). Recurrent respiratory papillomatosis: altered CD8(+) T-cell subsets and T(H)1/T(H)2 cytokine imbalance. Clin Immunol.

[B6] Lowy DR, Howley PM, Knipe DM, Howley PM, Griffin DE, Lamb RA, Martin MA, Roizman B, Straus SE (2001). Papillomaviruses. Fields virology.

[B7] Tindle RW, Frazer IH (1994). Immune response to human papillomaviruses and the prospects for human papillomavirus-specific immunization. Curr Top Microbiol Immunol.

[B8] Frazer IH, Thomas R, Zhou J, Leggatt GR, Dunn L, McMillan N, Tindle RW, Filgueira L, Manders P, Barnard P, Sharkey M (1999). Potential strategies utilized by papillomavirus to evade host immunity. Immunological Reviews.

[B9] Selsted ME, Ouellettee AJ (2005). Mammalian defensins in the antimicrobial immune response. Nat Immunol.

[B10] Lehrer RI, Lichtenstein AK, Ganz T (1993). Defensins: antimicrobial and cytotoxic peptides of mammalian cells. Annu Rev Immunol.

[B11] Valore EV, Park CH, Quayle AJ, Wiles KR, McCray PM, Ganzet T (1998). Human betadefensin1: an antimicrobial peptide of urogenital tissues. J Clin Invest.

[B12] Schnapp D, Harris A (1998). Antibacterial peptides in bronchoalveolar lavage fluid. Am J Respir Cell Mol Biol.

[B13] Lee SH, Lim HH, Lee HM, Choi JO (2000). Expression of human beta defensin-1 mRNA in human nasal mucosa. Acta Otolaryngol.

[B14] Krisanaprakornkit S, Weinberg A, Perez CN, Dale BA (1998). Expression of the peptide antibiotic human betadefensin1 in cultured gingival epithelial cells and gingival tissue. Infect Immun.

[B15] Mathews M, Jia HP, Guthmiller JM, Losh G, Graham S, Johnson GK, Tack BF, McCray PB (1999). Production of beta defensin antimicrobial peptides by the oral mucosa and salivary glands. Infect Immun.

[B16] Ganz T (2003). Defensins: antimicrobial peptides of innate immunity. Nat Rev Immunol.

[B17] Lehrer RI (2004). Primate defensins. Nat Rev Microbiol.

[B18] Quinones-Mateu ME, Lederman MM, Feng Z, Chakraborty B, Weber J, Rangel HR, Marotta ML, Mirza M, Jiang B, Kiser P, Medvik K, Sieg SF, Weinberg A (2003). Human epithelial beta-defensins 2 and 3 inhibit HIV-1 replication. AIDS.

[B19] Gropp R, Frye M, Wagner TO, Bargon J (1999). Epithelial defensins impair adenoviral infection: implication for adenovirus mediated gene therapy. Hum Gene Ther.

[B20] Yang D, Chertov O, Bykovskaia SN, Chen Q, Buffo MJ, Shogan U, Anderson M, Schröder JM, Wang JM, Howard OMZ, Oppenheim JJ (1999). β-defensins: linking innate and adaptive immunity through dendritic and T cell CCR6. Science.

[B21] Matthews K, Leong CM, Baxter L, Inglis E, Yun K, Backstrom BT, Doorbar J, Hibma M (2003). Depletion of Langerhans cells in human papillomavirus type 16-infected skin is associated with E6-mediated down regulation of E-cadherin. J Virol.

[B22] Lenz P, Day PM, Pang YS, Frye SA, Jensen PN, Lowy DR, Schiller JT (2001). Papillomavirus-Like Particles Induce Acute Activation of Dendritic Cells. J Immunol.

[B23] Yang R, Wheeler CM, Chen X, Uematsu S, Takeda K, Akira S, Pastrana DV, Viscidi RP, Roden RBS (2005). Papillomavirus capsid mutation to escape dendritic cell-dependent innate immunity in cervical cancer. J Virol.

[B24] Offringa R, de Jong A, Toes RE, van der Burg SH, Melief CJ (2003). Interplay between human papillomaviruses and dendritic cells. Curr Top Microbiol Immunol.

[B25] Arndt U, Wennemuth G, Barth P, Nain M, Al-Abed Y, Meinhardt A, Gemsa D, Bacher M (2002). Release of Macrophage Migration Inhibitory Factor and CXCL8/Interleukin-8 from Lung Epithelial Cells Rendered Necrotic by Influenza A Virus Infection. J Virol.

[B26] Schaefer TM, Fahey JV, Wright JA, Wira CR (2005). Innate Immunity in the Human Female Reproductive Tract: Antiviral Response of Uterine Epithelial Cells to the TLR3 Agonist Poly (I:C). J Immunol.

[B27] Lee SH, Kim JE, Lim HH, Lee HM, Choi JO (2002). Antimicrobial defensin peptides of the human nasal mucosa. Ann Otol Rhinol Laryngol.

[B28] Yasin B, Wang W, Pang M, Cheshenko N, Hong T, Waring AJ, Herold BC, Wagar EA, Lehrer RI (2004). Theta defensins protect cells from infection by herpes simplex virus by inhibiting viral adhesion and entry. J Virol.

[B29] Zhang L, Yu W, He T, Yu J, Caffrey RE, Dalmasso EA, Fu S, Pham T, Mei J, Ho JJ, Zhang W, Lopez P, Ho DD (2002). Contribution of human alpha-defensin 1, 2, and 3 to the anti-HIV-1 activity of CD8 antiviral factor. Science.

[B30] Chang TL, Vargas J, DelPortillo A, Klotman ME (2005). Dual role of alpha-defensin-1 in anti-HIV-1 innate immunity. Journal of Clinical Investigation.

[B31] Leikina E, Delanoe-Ayari H, Melikov K, Cho MS, Chen A, Waring AJ, Wang W, Xie Y, Loo JA, Lehrer RL, Chernomordik LV (2005). Carbohydrate-binding molecules inhibit viral fusion and entry by crosslinking membrane glycoproteins. Nat Immunol.

[B32] Buck CB, Day PM, Thompson CD, Lubkowski J, Lu W, Lowy DR, Schiller JT (2006). Human alpha-defensins block papillomavirus infection. PNAS.

[B33] Gravitt PE, Peyton CL, Alessi TQ, Wheeler CM, Coutlee F, Hildesheim A, Schiffman MH, Scott DR, Apple RJ (2000). Improved Amplification of Genital Human Papillomaviruses. J Clin Microbiol.

[B34] Gravitt PE, Peyton CL, Apple RJ, Wheeler CM (1998). Genotyping of 27 Human Papillomavirus Types by Using L1 Consensus PCR Products by a Single-Hybridization, Reverse Line Blot Detection Method. J Clin Microbiol.

[B35] Livak KJ, Schmittgen TD (2001). Analysis of relative gene expression data using real-time quantitative PCR and the 2(-Delta Delta C(T)) Method. Methods.

